# Mixed Cutaneous Infection Caused by *Mycobacterium szulgai* and *Mycobacterium intermedium* in a Healthy Adult Female: A Rare Case Report

**DOI:** 10.1155/2015/607519

**Published:** 2015-02-18

**Authors:** Amresh Kumar Singh, Rungmei S. K. Marak, Anand Kumar Maurya, Manaswini Das, Vijaya Lakshmi Nag, Tapan N. Dhole

**Affiliations:** ^1^Department of Microbiology, BRD Medical College, Gorakhpur, Uttar Pradesh 273013, India; ^2^Department of Microbiology, Sanjay Gandhi Post Graduate Institute of Medical Sciences, Lucknow 226014, India; ^3^Department of Microbiology, All India Institute of Medical Sciences, Jodhpur 342005, India

## Abstract

Nontuberculous mycobacteria (NTMs) are ubiquitous and are being increasingly reported as human opportunistic infection. Cutaneous infection caused by mixed NTM is extremely rare. We encountered the case of a 46-year-old female, who presented with multiple discharging sinuses over the lower anterior abdominal wall (over a previous appendectomy scar) for the past 2 years. Microscopy and culture of the pus discharge were done to isolate and identify the etiological agent. Finally, GenoType Mycobacterium CM/AS assay proved it to be a mixed infection caused by *Mycobacterium szulgai* and *M. intermedium*. The patient was advised a combination of rifampicin 600 mg once daily, ethambutol 600 mg once daily, and clarithromycin 500 mg twice daily to be taken along with periodic follow-up based upon clinical response as well as microbiological response. We emphasize that infections by NTM must be considered in the etiology of nonhealing wounds or sinuses, especially at postsurgical sites.

## 1. Introduction

Nontuberculous mycobacteria (NTMs) also known as atypical mycobacteria or mycobacteria other than tuberculosis (MOTT) have been recognized since the late 19th century but being opportunists, these organisms did not gain much importance for a long time. However, presently the recovery of NTM from clinical specimens from sites where they have been proved to cause infections (called “other mycobacterioses”) is of concern to microbiologists and physicians alike [1]. NTMs are ubiquitous organisms that are readily isolated from soil, water, domestic and wild animals, milk, and other items [2]. NTM on isolation was believed to represent environmental contamination or colonization for a long time; it was only during the late 1950s that NTMs were recognized as potential pathogens [3]. Nearly every pathogenic species of NTM may cause skin and soft tissue infections, but rapidly growing mycobacteria (*M. fortuitum*,* M. chelonae*, and* M. abscessus*),* M. marinum* and* M. ulcerans*, are the ones most commonly involved. Many of these cutaneous mycobacteria, such as the rapidly growing mycobacteria,* M. marinum, M. avium* complex,* M. kansasii*, and* M. xenopi*, are distributed worldwide [4]. The incidence of NTM infection has increased manifold, so much so that these infections currently account for 10%–15% of all mycobacterial infections [5, 6]. While the* Mycobacterium avium *complex (MAC) and* M. kansasii* are responsible for more than 90% of cases of nontuberculous mycobacterial infection, number of clinically important* Mycobacterium* species have rapidly increased and now include species such as* M. szulgai* [7].* M. szulgai* has been recovered from environmental sources, including a snail, aquarium water, swimming pool water, and tropical fish [8]. The environment is the suspected source of human NTM infection [9, 10].* M. szulgai* is an uncommon mycobacterial pathogen of humans [11]. It causes pulmonary disease resembling the common type of* M. tuberculosis* infection, as well as extrapulmonary infections. Cutaneous infection caused by* M. szulgai* was reported in one case which was seen after bone marrow transplantation [12], but mixed infection caused by two NTM species is extremely rare.* M. intermedium* is an intermediate to slow growing* Mycobacterium* that was first reported in 1993 [13]. We present here a case of an apparently immunocompetent patient with a history of previous surgery presenting with a mixed infection of the skin and soft tissue caused by* M. szulgai* and* M. intermedium*.

## 2. Case Report

A 46-year-old nondiabetic woman from a middle-class family, presented with discharging sinuses over the lower anterior abdominal wall for the past 2 years. There was no rise of temperature or weight loss. Her incision wound from laparoscopic appendectomy in 1998 did not heal properly and the surgical site was reopened after 1 month. There was no healing of the incision wound after reexcision. After that the whole area started to become erythematous and indurated and later on a keloid started developing over the scar. A large sinus developed over the operation scar and the other stitch sites also developed into sinuses discharging pus. However, there was no history of cough, haemoptysis, breathlessness, and anorexia. There was no history of intake of any immunosuppressive drugs/corticosteroids, any local trauma/injury, or tuberculosis. She had been treated off and on without having any definitive microbiological diagnosis with multiple injectable and oral antibiotics before consultation at this hospital. She did not have any contact with tuberculosis patients.

**Figure 1:**
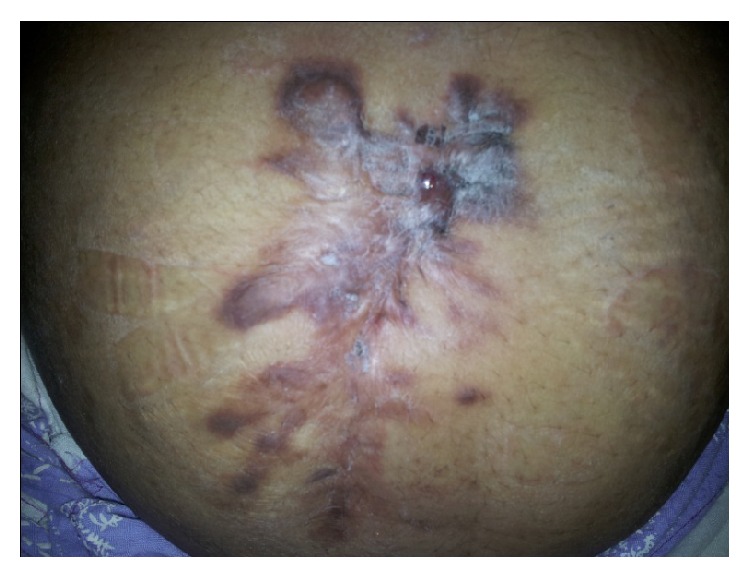
Clinical photograph showing discharging (serosanguinous as white arrow) sinuses present over previous-surgical scar with scarring and keloid formation on lower anterior abdominal wall.

**Figure 2:**
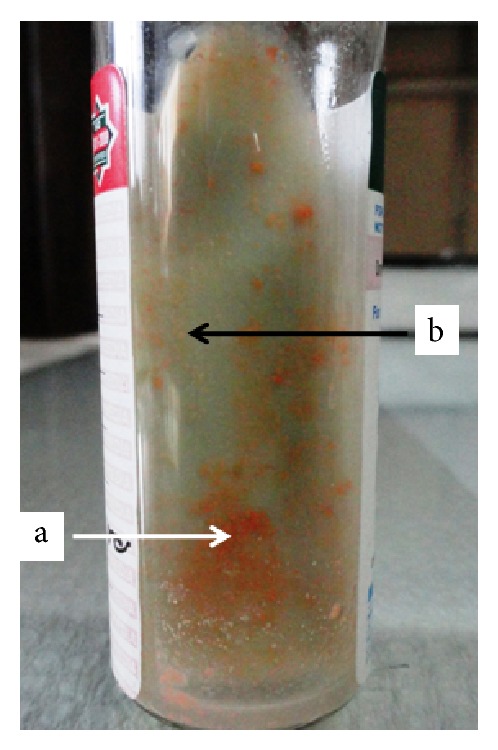
Showing numerous dry, rough, pigmented colonies of* M. szulgai* (a: bright orange as white arrow) and* M. intermedium *(b: pale/light yellow as black arrow) over Lowenstein-Jensen media slant.

On examination, a previous-surgical scar was present over which a large keloid had formed, on the lower anterior abdominal wall in the midline (although a little more towards the right). There was no apparent mass over the abdomen, but 6 sinus openings were noted over the lower anterior abdominal wall (Figure 1). At the time of presentation a single sinus was discharging pus. On palpation, tenderness was noted over and around the sinuses. The sinus opening was bluish, wide, and undermined and it was discharging a serosanguinous exudate admixed with pus. Complete haemogram and clinical chemistry were within normal limits including blood sugar. HIV ELISA negative by two kits, C-reactive protein normal and the ESR, was peaked at 22 mm. Pus from the discharging sinus was collected and processed using standard procedures. Gram stained smear showed plenty of pus cells with Gram variable bacilli (many of which were Gram positive) and on Ziehl–Neelsen staining numerous acid fast bacilli could be observed. The discharge was cultured on blood agar, MacConkey medium, and Lowenstein-Jensen medium and inoculated into a BacT/ALERT MP bottle, which was incubated in the BacT/ALERT 3D system (bioMerieux, USA). The blood agar and MacConkey medium were incubated under aerobic conditions at 37°C. The blood agar plate showed numerous dry, small nonhemolytic colonies after 72 hours but there was no growth on the MacConkey medium. The BacT/ALERT MP showed positive growth in 5 days for acid fast bacilli, which on further subculture onto L-J medium gave dry, rough, pigmented, and yellow to orange colonies after one-week period (Figure 2). They were found to be scotochromogenic at 37°C. Speciation for the isolate was confirmed by the GenoType Mycobacterium CM/AS assay based on Mycobacterium DNA strip technology (Hain Lifescience, Nehren, Germany), [14] following manufacturer's instructions. It showed a mixed infection caused by two NTM species, that is,* Mycobacterium szulgai* and* M. intermedium*. To confirm microbiological result, culture from repeat sample after one week showed the same dual infection caused by* Mycobacterium szulgai* and* M. intermedium* by the GenoType Mycobacterium CM/AS assay. Drug susceptibility testing (DST) was performed by standard disk diffusion method over Middlebrook 7H10 agar supplemented with OADC (oleic acid, dextrose, and citrate) and the following results were obtained: sensitive to linezolid, levofloxacin, clarithromycin, amikacin, and ciprofloxacin and found to be resistant to cotrimoxazole. DST for 1st and 2nd line antitubercular drugs were done by the GenoType MTBDR*plus* and MTBDR*sl* assay (Hain Lifescience, Nehren, Germany) which showed resistance to isoniazid and sensitive to rifampicin, fluoroquinolones, aminoglycosides/cyclic peptides, and ethambutol.

The patient was advised a combination of rifampicin 600 mg once daily, ethambutol 600 mg once daily, and clarithromycin 500 mg twice daily to be taken along with periodic follow-up based upon clinical response as well as microbiological response.

She was advised to continue the therapy and when she presented to the hospital after one month, there was no pus discharge from the sinus. She was advised to continue the therapy and report to hospital after another month. Again after one month of therapy, there was no discharge present and the sinus was almost completely healed. Thereafter, she was advised to continue the same therapy for another two months. Thereafter, patient was completely all right with no such discharge after completion of two-month antibiotic therapy.

## 3. Discussion

Nontuberculous mycobacteria (NTMs) can be classified by their growth rate as slowly growing and rapidly growing species, by pigment production (pigmented and nonpigmented species) and optimal growth temperature requirement.* M. marinum*,* M. kansasii, M. avium-intracellulare, M intermedium*, and* M. szulgai* are examples of slow-growing mycobacteria.* M. fortuitum, M. chelonae, *and* M. abscessus* are examples of rapidly growing mycobacteria [4]. Diagnosis relies upon clinical presentations, microscopy, microbiological culture, and molecular detection of mycobacterial DNA to confirm species identification. NTMs have had a strong impact on human populations in both developing and industrialized countries [2]. Cutaneous infections caused by NTMs are still uncommon but their relative importance has changed during last decade and still further changes are expected.* M. szulgai *is a slow growing, scotochromogenic NTM, first identified in 1972 by a Polish microbiologist T Szulg.* M. intermedium* was first identified in 1993 and classified as a photochromogen, usually causing chronic bronchitis, dermatitis, and sometimes pulmonary infections. The name “intermedium” was used to denote its intermediate phylogenetic position between the slowly and rapidly growing mycobacteria [15]. The infection caused by* M. szulgai* is very rare and this bacterium accounts for 0.5% of all isolates obtained from human patients with NTM infection [16, 17].* M. szulgai* when isolated is thought to be clinically significant unless proven otherwise and treatment should always be considered [18]. Only 27 cases of* M. szulgai* infection have been reported between 1989 and 2008 from Japan, most of which involved preexisting disease [19]. Cultures yielding* M. szulgai *usually have a pathologic significance, because this bacterium is rarely recovered from the environment [17, 18]. The acquisition of* M. szulgai* from the local environment especially water was already proven by different previous studies [9, 10, 20]. The sensitivity and specificity of the GenoType Mycobacterium CM strip have been shown to be 97.0–98.9% and 88.9–92.4% and of the AS strip 99.3-99.4 and 99.4–100%, respectively [21, 22]. Consequently, even one positive culture under appropriate clinical circumstances may suffice for diagnosing* M. szulgai* infection. In the present case, cultures of the sinus exudate from the anterior abdominal wall revealed mixed infection caused by* M. szulgai* and* M. intermedium*. The source of infection in the patient fits the pattern potentially resulting from introduction of mycobacteria during surgery, because it was present at the site of laparoscopic ports. However, the source of the infection could not be proved in this case because surgery was done in a private hospital setting and could not be accessed by the authors.* M. szulgai* can cause chronic nonhealing skin and soft tissue infection through iatrogenic spread.* M. intermedium *has been known to cause granulomatous dermatitis in a patient with hot tub exposure and appeared to be refractory to appropriate antimicrobial therapy because of repeated exposure to contaminated water [23].* M. szulgai* infection causing nonhealing sinus is extremely rare but can be successfully treated with a combination of different antibiotics and antitubercular drugs.

Our case report highlights the paramount importance of eliciting adequate medical history in the diagnosis of NTM infection. The patient failed to respond to seemingly appropriate antibiotic therapy over a 2-year period due to the lack of definite microbiological diagnosis. In this case, the patient had undergone surgery for appendicitis and at that time she presented with nonhealing wound and was treated with off and on multiple antibiotics. The discharging sinuses developed later at the site of previous surgery and during that time no attempt was made for appropriate diagnosis. The reservoir for many mycobacterial species is generally municipal and hospital water supplies. These mycobacterial species are incredibly hardy and able to grow in tap water and distilled water, thrive at temperatures of 45°C or above (especially* M. xenopi* and* M. avium *complex), and resist the activity of organomercurials, chlorine, 2% concentrations of formaldehyde, and alkaline glutaraldehyde [24]. Perhaps in our patient, the nontuberculous mycobacteria were transmitted from a contaminated environmental source by the direct cutaneous route. Nevertheless, even in a healthy individual exposure to saprophytic mycobacteria may lead to infections, as described for* M. kansasii*. It is difficult to recognize and treat NTM infections, especially mixed infections caused by unusual pathogens like* M. szulgai* and* M. intermedium*. A high index of suspicion is warranted in these cases and prompt treatment should be initiated once adequate material for diagnosis has been secured.

We insist on strict adherence to standard sterilization procedures for surgical instruments, medical equipment, skin-marking solutions, water supplies, and proper preoperative skin cleansing, since all are important factors with a definite influence in the initiation of these infections. Finally, mycobacteria should be included in the differential diagnosis of nonhealing wound and soft tissue infections after surgical procedures.
